# Climate change, extreme events, and increased risk of salmonellosis: foodborne diseases active surveillance network (FoodNet), 2004-2014

**DOI:** 10.1186/s12940-021-00787-y

**Published:** 2021-09-18

**Authors:** Michele E. Morgado, Chengsheng Jiang, Jordan Zambrana, Crystal Romeo Upperman, Clifford Mitchell, Michelle Boyle, Amy R. Sapkota, Amir Sapkota

**Affiliations:** 1 Maryland Institute for Applied Environmental Health, University of Maryland School of Public Health, 2234F SPH Building #255, College Park, MD 20742 USA; 2 Aclima, Inc., San Francisco, CA USA; 3 Maryland Department of Health, Prevention and Health Promotion Administration, Baltimore, MD USA

**Keywords:** *Salmonella*, Foodborne illness, Climate change, Extreme heat, Extreme precipitation

## Abstract

**Background:**

Infections with nontyphoidal *Salmonella* cause an estimated 19,336 hospitalizations each year in the United States. Sources of infection can vary by state and include animal and plant-based foods, as well as environmental reservoirs. Several studies have recognized the importance of increased ambient temperature and precipitation in the spread and persistence of *Salmonella* in soil and food. However, the impact of extreme weather events on *Salmonella* infection rates among the most prevalent serovars, has not been fully evaluated across distinct U.S. regions.

**Methods:**

To address this knowledge gap, we obtained *Salmonella* case data for *S.* Enteriditis, *S.* Typhimurium, *S.* Newport, and *S.* Javiana (2004-2014; n = 32,951) from the Foodborne Diseases Active Surveillance Network (FoodNet), and weather data from the National Climatic Data Center (1960-2014). Extreme heat and precipitation events for the study period (2004-2014) were identified using location and calendar day specific 95^th^ percentile thresholds derived using a 30-year baseline (1960-1989). Negative binomial generalized estimating equations were used to evaluate the association between exposure to extreme events and salmonellosis rates.

**Results:**

We observed that extreme heat exposure was associated with increased rates of infection with *S.* Newport in Maryland (Incidence Rate Ratio (IRR): 1.07, 95% Confidence Interval (CI): 1.01, 1.14), and Tennessee (IRR: 1.06, 95% CI: 1.04, 1.09), both FoodNet sites with high densities of animal feeding operations (e.g., broiler chickens and cattle). Extreme precipitation events were also associated with increased rates of *S.* Javiana infections, by 22% in Connecticut (IRR: 1.22, 95% CI: 1.10, 1.35) and by 5% in Georgia (IRR: 1.05, 95% CI: 1.01, 1.08), respectively. In addition, there was an 11% (IRR: 1.11, 95% CI: 1.04-1.18) increased rate of *S*. Newport infections in Maryland associated with extreme precipitation events.

**Conclusions:**

Overall, our study suggests a stronger association between extreme precipitation events, compared to extreme heat, and salmonellosis across multiple U.S. regions. In addition, the rates of infection with *Salmonella* serovars that persist in environmental or plant-based reservoirs, such as *S.* Javiana and *S.* Newport, appear to be of particular significance regarding increased heat and rainfall events.

**Supplementary Information:**

The online version contains supplementary material available at 10.1186/s12940-021-00787-y.

## Introduction

There are an estimated 9.4 million cases of foodborne illness each year in the United States [1]. Nontyphoidal *Salmonella* spp. is a leading cause of U.S. foodborne illnesses, resulting in 19,336 hospitalizations and an estimated 378 deaths each year [1]. Infections with nontyphoidal *Salmonella* are frequently associated with contaminated poultry, meat products, eggs, and fresh produce [2–5]; however, animal sources of contamination can be markedly different by state [6]. Symptoms of *Salmonella* infection, or salmonellosis, can manifest from 12 to 72 hours after exposure and are characterized by an acute onset of fever, diarrhea, and abdominal cramps [7]. Gastrointestinal illness caused by *Salmonella* usually self resolves within a few days, but vulnerable populations (e.g., children under 5 years old, elderly persons, and immunocompromised patients) are more likely to develop severe complications such as bacteremia [7, 8].

There are over 2,500 recognized *Salmonella enterica* serovars [9] and each one contains a different variation of cell-surface carbohydrates and flagellar proteins, known as O and H antigens [10]. These serovars may dominate a wide range of ecological niches and can also exhibit varying degrees of virulence [11]. The *S. enterica* serovars Enteritidis and Typhimurium are responsible for most of the salmonellosis cases across the globe, most likely due to their high rates of infection in chickens [10]. Currently, the serovars *S.* Enteritidis, *S.* Typhimurium, *S.* Newport, and *S.* Javiana account for over 50% of the fully serotyped isolates characterized in the U.S. [5].

Several recent studies have observed an association between food and waterborne disease occurrence and increased ambient temperature and precipitation [12–22]. This is of particular importance given that current increasing trends in the frequency, intensity and duration of extreme heat and precipitation events are projected to grow in response to climate change [23]. These changes have the potential to exacerbate bacterial proliferation, as well as soil and water contamination, and present an added challenge to the prevention of foodborne illness [24]. The increased spread and persistence of *Salmonella* spp. associated with extreme weather events is especially concerning in food commodities that are often eaten raw, such as fruits and vegetables [24, 25].

A recent study by our group demonstrated the impact of extreme temperature and precipitation events on the risk of *Salmonella* infection in the State of Maryland [16]. We described a 4.1% and a 5.6% increase in salmonellosis risk associated with a 1-unit increase in extreme temperature and precipitation events, respectively [16]. This risk was more pronounced in coastal versus non-coastal areas; however, our findings were limited to the State of Maryland. The present study builds upon our previous work, evaluating the impact of extreme temperature and precipitation events on the risk of salmonellosis with the four most common serovars (*S*. Enteritidis, *S.* Typhimurium, *S.* Newport, and *S.* Javiana) across multiple U.S regions.

## Methods

### Data sources

*Salmonella* case data were obtained from the Foodborne Diseases Active Surveillance Network (FoodNet), a collaboration between the Centers for Disease Control and Prevention (CDC), 10 state health departments, the US Department of Agriculture’s Food Safety and Inspection Service (USDA-FSIS), and the US Food and Drug Administration (FDA). FoodNet sites are located throughout the country and include ten states that represent roughly 15% of the total U.S. population. These FoodNet sites conduct active, population-based surveillance on laboratory-confirmed infections that are caused by nine pathogens commonly transmitted through food, including *Salmonella.* For this study, we limited our analyses to culture-confirmed *Salmonella* cases from the seven FoodNet sites with active surveillance across all counties (Connecticut, Georgia, Maryland, Minnesota, New Mexico, Oregon, and Tennessee). In addition, we restricted the analyses to reported cases of the following four predominant *Salmonella* serovars between 2004 and 2014: Enteriditis, Javiana, Newport, and Typhimurium. We defined a case as an individual whose biological specimen (stool, blood, or other) was culture confirmed for the presence of *Salmonella*, regardless of symptoms or date of onset.

We obtained age, sex, and race/ethnicity data from the 2010 Census of Population and Housing, Summary File 1 and poverty data from the American Community Survey 2006-2010 [26]. These data were downloaded at the county level from the Census website and used to calculate county level percentages of 1) people in the age groups <5, 5-17, 18-64, and ≥65; 2) individuals living below the poverty level in 2010; 3) populations of individual races; and 4) males and females. Concentrated animal feeding operation data were obtained from the 2007 U.S. Census of Agriculture, National Agriculture Statistics Service [27].

### Coastal county definition

Counties were classified as coastal or non-coastal based on National Oceanic and Atmospheric Administration (NOAA) definitions outlined in its coastal assessment framework [28]. Specifically, NOAA defines a county as a coastal county if: “1) at least 15% of the county’s total land area is located within a coastal watershed, or 2) a portion of or an entire county accounts for at least 15% of a coastal U.S. Geological Survey 8-digit cataloging unit” [28].

### Weather data and extreme heat/precipitation events

We obtained daily weather data from the National Climatic Data Center website for the 1960-2014 period, including daily maximum temperature (TMAX) and precipitation (PRCP) [29]. Details regarding identification of extreme heat and precipitation events have been described previously [16, 30]. In brief, we used daily TMAX and PRCP for the 1960-1989 period to compute calendar day and location (county) specific 95^th^ percentile thresholds for TMAX and PRCP, which are referred to as Extreme Temperature Threshold 95^th^ percentile (ETT_95_) and Extreme Precipitation Threshold 95^th^ percentile (EPT_95_), respectively. Daily PRCP and TMAX values during the study period for which we have the FoodNet data (2004-2014) were compared to their respective calendar day and location specific 95^th^ percentile thresholds and assigned a value of “1” if they exceeded the thresholds, and “0” otherwise. Days exceeding the TMAX thresholds were identified as extreme heat events and those exceeding the PRCP thresholds were identified as extreme precipitation events. The rationale behind the use of extreme events as exposure metric instead of continuous temperature/precipitation is their relevance in the context of climate change and existing literature linking weather variables with salmonellosis.

### Statistical model

We used negative binomial Generalized Estimating Equations (GEE) [31, 32] ) to investigate the relationship between exposure (monthly count of extreme weather events) and outcome (monthly count of salmonellosis cases) to account for overdispersion and repeated nature of outcome measure. First, we ran an overall analysis, adjusting for potential confounders including poverty status, age, sex, and race. We then performed stratified analyses by race (Non-Hispanic White, Non-Hispanic Black), degree of urbanization (urban, suburban, rural), density of broiler chicken operations (high, moderate, low), and geographic location (coastal counties, non-coastal counties). The PROC GENMOD command with REPEATED statement was used for controlling the autocorrelation of repeated measurements within each county. All statistical analyses were performed using SAS 9.4 (Cary, NC USA).

## Results

Between 2004 and 2014, 32,951 cases of culture-confirmed *Salmonella* infection from serovars Enteritidis, Javiana, Newport, and Typhimurium were reported to FoodNet by Connecticut, Georgia, Maryland, Minnesota, New Mexico, Oregon, and Tennessee (Table 1).

**Table 1:** Characteristics of FoodNet reported cases of *Salmonella* serovar Enteritidis, Javiana, Newport, and Typhimurium by the states of Connecticut, Georgia, Maryland, Minnesota, New Mexico, Oregon, and Tennessee between 2004-2014

Characteristic	% Population	Salmonella serovar (# cases, # cases per 10,000)			
		Enteritidis	Javiana	Newport	Typhimurium
**Age**					
0 to 4	6.5	1510 (5.5)	2391 (8.6)	2378 (8.6)	2877 (10.4)
5 to 17	17.5	1678 (2.3)	967 (1.3)	973 (1.3)	1866 (2.5)
18 to 64	63.3	6281 (2.3)	1860 (0.7)	3094 (1.1)	3412 (1.3)
≥ 65	12.6	1167 (2.2)	587 (1.1)	1002 (1.9)	871 (1.6)
Unreported	NA	17	4	6	10
**Gender**					
Female	51.0	5531 (2.5)	2970 (1.4)	3899 (1.8)	4353 (2.0)
Male	49.0	5098 (2.4)	2823 (1.4)	3534 (1.7)	4668 (2.2)
Unreported	NA	24	16	20	15
**Race/Ethnicity**					
Non-Hispanic Black	17.4	1965 (2.7)	787 (1.1)	701 (0.9)	1363 (1.8)
Non-Hispanic White	66.0	5324 (1.9)	2784 (1.0)	3977 (1.4)	4407 (1.6)
Hispanic	10.2	678 (1.6)	366 (0.8)	484 (1.1)	903 (2.1)
Other	6.4	319 (1.2)	87 (0.3)	174 (0.6)	311 (1.1)
Unreported	NA	2367	1785	2117	2052
**% Poverty**					
High	17.4	2421 (3.3)	2468 (3.3)	2807 (3.8)	2102 (2.8)
Median	35.1	2884 (1.9)	1766 (1.2)	2256 (1.5)	3071 (2.1)
Low	47.4	5348 (2.7)	1575 (0.8)	2390 (1.2)	3863 (1.9)
**Urbanization**					
Rural	15.6	1537 (2.3)	1714 (2.6)	2095 (3.2)	2005 (3.0)
Suburb	30.2	2768 (2.2)	2533 (2.0)	2669 (2.1)	2769 (2.2)
Urban	54.2	6348 (2.8)	1562 (0.7)	2689 (1.2)	4262 (1.8)
**Broiler Chicken Operations**					
High	32.5	3145 (2.3)	624 (0.5)	1235 (0.9)	2743 (2.0)
Moderate	37.2	3864 (2.4)	2416 (1.5)	3137 (2.0)	3347 (2.1)
Low	30.3	3644 (2.8)	2769 (2.1)	3081 (2.4)	2946 (2.3)
**Region**					
Coastal	33.7	4822 (3.4)	2078 (1.4)	2395 (1.7)	2599 (1.8)
Non-Coastal	66.3	5831 (2.1)	3731 (1.3)	5058 (1.8)	6437 (2.3)

Among the 7 FoodNet sites analyzed, most *S*. Enteritidis (5.5 per 10,000), *S.* Javiana (8.6), *S.* Newport (8.6) and *S.* Typhimurium (10.4) cases occurred among those aged 0-4 years old (Table 1). Salmonellosis cases across all serovars were similarly distributed between males and females but showed some variability among race/ethnicity groups, with the highest observed cases per 10,000 in Non-Hispanic Black populations (2.7) for *S*. Enteriditis (Table 1). *Salmonella* cases for all serovars, *S.* Enteriditis (3.3), *S.* Javiana (3.3), *S.* Newport (3.8), and *S.* Typhimurium (2.8), were more frequently reported in counties with higher poverty rates (Table 1). In terms of other characteristics, salmonellosis cases were reported more frequently in rural settings for most serovars, except for *S.* Enteriditis(2.8 per 10,000 in urban), and mostly inlow-density broiler chicken operation areas (Table 1).

**Fig. 1:**
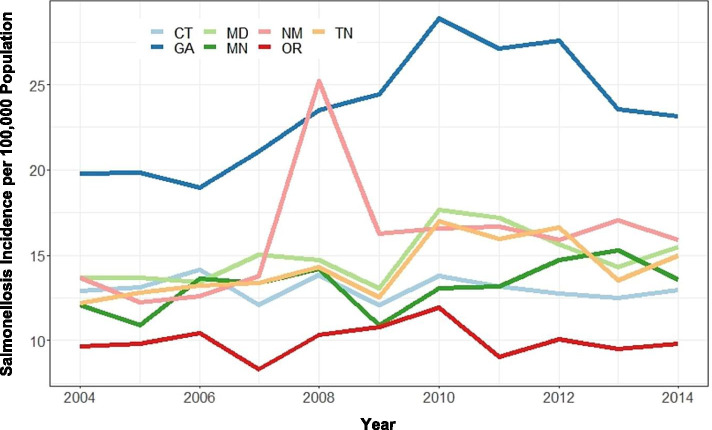
Incidence of Salmonella infection per 100,000 population for all serovars by year and by state during 2004-2014. The FoodNet sites are Connecticut (CT); Maryland (MD); New Mexico (NM); Tennessee (TN); Georgia (GA); Minnesota (MN); and Oregon (OR)

The average incidence of salmonellosis (across all reported serovars) among all seven FoodNet sites was highest in Georgia (23.4 cases per 100,000 population) and lowest in Oregon (10.0 cases per 100,000 population) (Fig. 1). New Mexico had the second highest average incidence of salmonellosis (16.0 cases per 100,000 population), while the remainder of the states averaged roughly 13.8 cases per 100,000 population (Fig. 1).

**Fig. 2:**
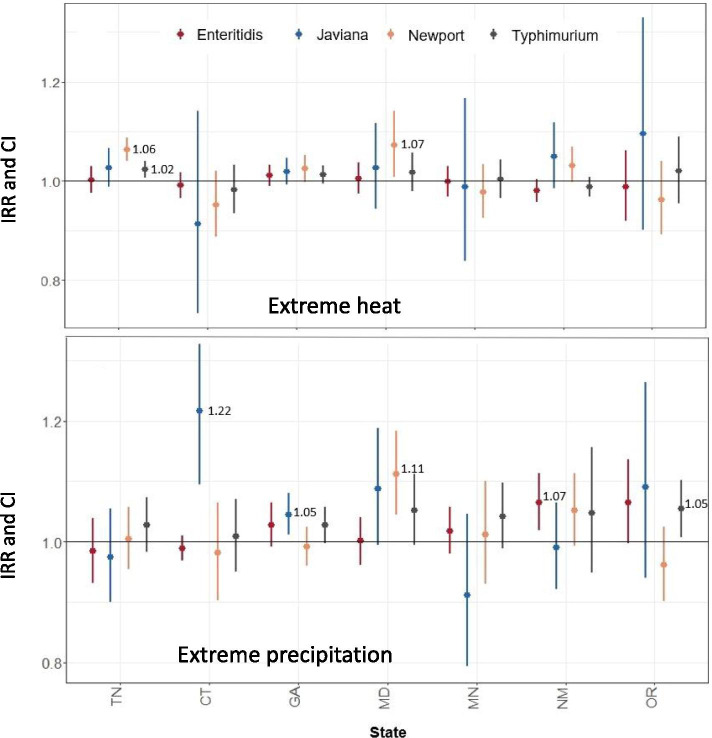
Salmonella incidence rate ratios (IRRs) and 95% confidence intervals (CIs) for exposures to extreme temperature (ETT_95_ exceedance: panel **A**) and precipitation (EPT_95_ exceedance: panel **B**) events across 7 FoodNet sites by serovar (Enteritidis, Javiana, Newport, and Typhimurium)

Extreme heat and precipitation related risk of salmonellosis varied considerably between FoodNet sites and across serovars (Fig. 2). For instance, extreme heat exposure was associated with a statistically significant increased risk of infection with *S.* Newport in two out of seven FoodNet sites (Tennessee and Maryland) (Fig 2A). In Tennessee, we observed a 6% increase in risk of *S.* Newport infections (Incidence Rate Ratio (IRR): 1.06, 95% Confidence Interval (CI): 1.04-1.09) and in Maryland we observed a 7% increase in risk (IRR: 1.07, 95% CI: 1.01-1.14). Meanwhile, significant but less robust increases in the risk of *S.* Newport infections were observed in Georgia (IRR: 1.03, 95% CI: 1.00-1.05) and New Mexico (IRR: 1.03, 95% CI: 1.00-1.07) (Fig 2A).

Extreme precipitation events increased the risk of *S*. Javiana by 22% in Connecticut (IRR: 1.22, 95% CI: 1.10-1.35) and by 5% (IRR: 1.05, 95% CI: 1.01-1.08) in Georgia, while the increase in risk was borderline significant in Maryland (IRR: 1.09, 95% CI: 0.99-1.19) (Fig 2B). Similarly, extreme precipitation events were associated with an 11% increase in the risk of *S.* Newport (IRR: 1.11, 95% CI: 1.04-1.18) in Maryland, and a 7% increase in the risk of *S.* Enteritidis in New Mexico (IRR: 1. 07, 95% CI: 1.02-1.11). In Oregon, extreme precipitation was associated with increased rates of *S.* Typhimurium infections (IRR: 1.05, 95% CI: 1.01-1.10) and *S*. Enteriditis infections, although the latter was only borderline significant (IRR: 1.07, 95% CI: 1.00-1.14) (Fig 2B).

**Fig. 3:**
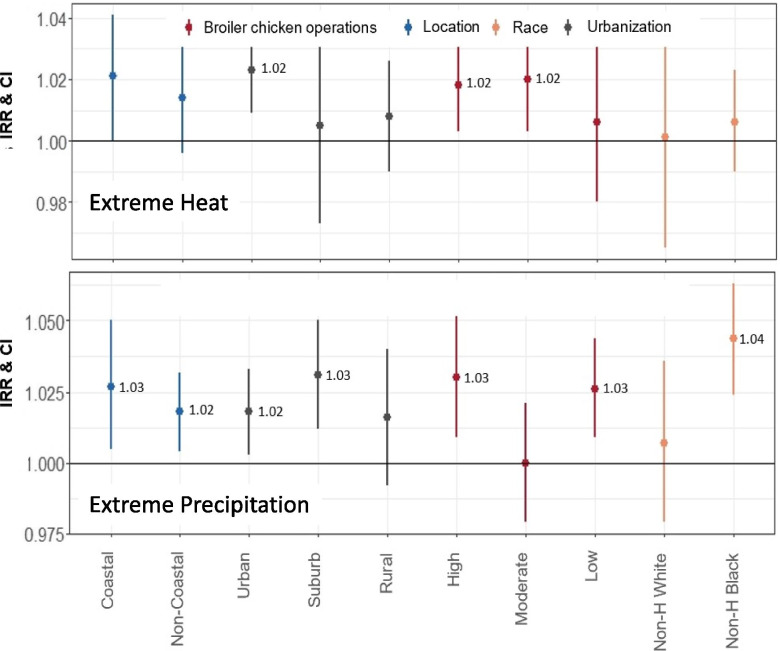
Salmonella incidence rate ratios (IRRs) and 95% confidence intervals (CIs) for exposures to extreme heat (ETT_95_ exceedance: panel **A**) and precipitation (EPT_95_ exceedance: panel **B**) events across 7 FoodNet sites by urbanization, race, coastal/non-coastal, and density of broiler chicken operations

We performed stratified analyses to examine whether the association between salmonellosis risk and extreme temperature and precipitation events varied by density of broiler chicken operations, geographic location, race, and urbanization level (Fig. 3). Extreme heat related salmonellosis risks were significant in urban counties (IRR: 1.02, 95% CI: 1.01-1.04) and counties with moderate to high density of broiler chicken operations (Fig. 3A). By comparison, extreme precipitation events increased salmonellosis incidence in both coastal and non-coastal counties, as well as urban and suburban locations, but not in rural areas. We also observed higher extreme precipitation-related salmonellosis risks among non-Hispanic Blacks (IRR: 1.04, 95% CI: 1.02-1.06) compared to non-Hispanic Whites (IRR: 1.01, 95% CI: 0.98-1.04) (Fig. 3B).

## Discussion

Several studies have observed that the prevalence of *Salmonella* infections is strongly influenced by changes in ambient temperature [12, 18, 22, 33–38], with higher rates often observed during the summer [12, 22, 39]. Multiple studies have also provided evidence that salmonellosis rates are positively associated with increased rainfall events [19, 22, 33, 40]. Moreover, there is growing evidence suggesting projected changes to climate [23] will affect the incidence of foodborne illness, including *Salmonella* infections [12, 16, 18, 24, 25, 41]. Our findings provide further evidence that increases in ambient temperature and precipitation levels, particularly in the form of extreme events, are associated with increases in rates of salmonellosis. Additionally, our results show that the response to this association is not uniform across different regions in the U.S., or among the four most prevalent *Salmonella* serovars.

The heterogeneity in this relationship is exemplified by *S.* Newport, which was associated with extreme heat events in 4 out of 7 FoodNet sites (Georgia, Maryland, New Mexico, and Tennessee), whereas higher extreme precipitation related risks for all four serovars were observed across a greater number of FoodNet sites. *Salmonella* infections can be serovar-specific and have been shown to differ geographically [42, 43], across age and gender [42], among race/ethnicity groups and by poverty levels [43, 44]. In addition, sources of infection and routes of transmission can vary among *Salmonella* serovars [4]. Previous studies have also found that *Salmonella* serovars may respond differently to extreme heat events [45] and that serovars with environmental reservoirs (such as *S.* Javiana) may be more heavily impacted by increased precipitation [19, 46]. The uneven response that we observed among the four serovars (*S.* Enteriditis, *S.* Javiana, *S*. Newport, and *S.* Typhimurium) regarding extreme heat and precipitation events, suggests that environmental reservoirs and exposure pathways for *Salmonella* infection may be of increased relevance.

The elevated rate of infection with *S.* Newport in Georgia, New Mexico, Tennessee, and Maryland associated with extreme heat exposure could reflect the serovar’s ability to contaminate a wide range of food commodities, especially fresh produce and dairy or beef cattle [4, 47], which represent diverse foods commonly grown in these states [27]. In addition, *S*. Newport has been shown to persist and survive in elevated temperatures inside fresh produce crops such as tomatoes [48]. These states are especially characterized by high densities of broiler chicken operations (Georgia and Maryland), and a greater presence of cattle (Tennessee) and dairy (New Mexico) operations in rural areas [27]. Previous works have documented occupational exposures to *Salmonella* spp. among workers in livestock operations [42, 48, 49], as well as the sustained proliferation of *Salmonella* in the environment when using untreated waste from animal agriculture [50, 51]. Animal waste can contaminate soil, surface waters and groundwater through runoff and has been shown to be a source of *Salmonella* [51]. In addition, studies have pointed to the persistence of *S.* Newport isolates in irrigation water and soil in the Mid-Atlantic region, which may act as long-term reservoirs of contamination [52, 53]. Moreover, it has been observed that *Salmonella* internalization within fresh produce might be more pronounced with extreme weather conditions, including drought from sustained periods of heat [54]. There are also concerns of increased dissemination of pathogens such as *Salmonella* from grazing cattle as elevated temperatures may cause them to harbor and shed bacteria at higher levels [24, 55, 56].

Extreme precipitation events can increase the frequency of human contact with contaminated water sources [57–60], and they can also increase the likelihood of fresh produce coming into contact with contaminated runoff [24, 61, 62]. This can be especially important for the dissemination and persistence of *Salmonella* serovars with natural reservoirs, such as *S.* Javiana [4, 19, 46, 63], as well as those frequently associated with plant-derived food commodities, such as *S.* Newport [4]. In our study we observed a significant increase in the risk of *S.* Javiana infections in the states of Connecticut and Georgia, as well as an elevated risk of *S*. Newport in Maryland, associated with extreme precipitation events.

Previous studies have shown the importance of environmental and wild animal reservoirs, particularly amphibians and reptiles, in explaining the greater distribution of *S*. Javiana infections in the southeastern U.S. [46, 63, 64]. Correspondingly, a positive association was found between *S*. Javiana infections and the percentage of wetland coverage in Georgia, which provides a habitat for many amphibian species [65]. In addition, Lee et al. [19] observed that the infection risk of *S.* Javiana increased in the coastal plain region in Georgia, following periods of extreme rainfall. This region is characterized by extensive agricultural production, where fresh produce and livestock may overlap, as well as a large percentage of aging wells and septic systems [19]. These conditions can increase the risk of human exposure to contaminated water sources, either through the consumption of contaminated produce and drinking water, or with recreational use [22, 33, 54, 57, 63]. In contrast, the northeastern state of Connecticut reported less *S*. Javiana infections than Georgia during our study period, but the risk of infection associated with extreme rainfall events was far more pronounced. Wetland coverage is considerably diminished in Connecticut [66] and agricultural production covers the smallest area of the included states [67]; however, approximately 23% of its population are served by private well systems [68] compared to the national estimated average of 13% [69]. This could underscore the importance of private well contamination following periods of excessive rainfall, as well as the persistence of *Salmonella* serovars, such as *S.* Javiana in the soil and water [53, 70, 71].

*S*. Newport has also been found to survive for extensive periods of time in soil and water [53, 70], particularly in moist organic soil [71] which is prevalent in Maryland [72]. Studies have found that both extreme heat and precipitation can lead to an increased prevalence of *Salmonella* in crops such as lettuce, either through internalization or transfer of pathogens with contaminated surface waters [54]. As noted earlier, the elevated risk of *S.* Newport in Maryland associated with both extreme heat and precipitation events could reflect its continued persistence and ability to disseminate over land and contaminate crops [52, 53]. Additionally, recreational water activities and drinking from contaminated private wells may be an important route of human exposure following periods of heavy rainfall [12, 19, 33, 63].

Overall, our study indicated a stronger association between extreme precipitation events and salmonellosis across the U.S., compared to extreme heat events. Previous works have described the role of excessive rainfall in the proliferation of *Salmonella* in the soil, water, and a range of food commodities [12, 19, 61, 62, 70, 73]. Furthermore, the elevated risk of *Salmonella* infection associated with poultry and livestock reservoirs, as observed with *S.* Typhimurium and *S*. Enteriditis [4], may be of special consideration even in states without large animal production facilities, such as Oregon and New Mexico [67]. Potential gastrointestinal disease vectors could include rodent populations [74], which have been shown to increase in semi-arid regions like New Mexico following periods of wetness [75]. Wild birds can also be infected with *Salmonella*, particularly *S*. Typhimurium, representing a potential risk of contamination to local waterways and increased human exposure [49].

The wide geographic area (7 states) covered in this study, along with the rich dataset of county-specific heat and precipitation events and *Salmonella* cases used to develop the exposure and outcome metrics, are key strengths of this study. A unique aspect of our study is the analysis of differences in incidence with respect to several major *Salmonella* serovars (*S.* Enteritidis, *S.* Javiana, *S.* Newport and *S.* Typhimurium). The wide range of serovar responses to the extreme events across the FoodNet sites highlights the important role that state and local public health departments must play in the response to nationally identified associations. Given the large toll that salmonellosis takes on public health and the exacerbating impacts of climate change on its incidence, it is important that public health adaptation measures take these issues under consideration.

Limitations of this study are that the data were aggregated at the county level which does not consider other factors that might influence observed associations between serovar incidence rates and specific locations. We also focused on the daily exceedance of the thresholds of precipitation and temperature and did not assess the magnitude of the exceedance itself. Furthermore, we relied on the date of a culture positive test to ascertain relevant exposure, rather than the date of symptom onset or potential infection, which were not available. Likewise, we did not have information related to specific outbreaks that might have contributed to the temporal clustering of the cases. It is also important to note that while FoodNet is an active surveillance system, the data are an imperfect measure of the true incidence of foodborne illness. Reported cases of laboratory confirmed salmonellosis can often understate the true incidence of infections due to underreporting and underdiagnosis, and the data may represent some of the most severe cases.

## Conclusions

Our findings indicate that the association between salmonellosis incidence and extreme weather events is heterogenous among the four most common *Salmonella* serovars, and across multiple U.S. regions. Moreover, the elevated rates of infections with *S*. serovars that dominate in natural or plant-based reservoirs (e.g., *S.* Javiana, and *S.* Newport) associated with extreme heat and rainfall events, merits special attention. These findings emphasize the need for public health related decisions to be crafted at the state and local levels to meet each region’s changing environmental and climatic conditions.

## Supplementary Information

**Additional file 1: Supplemental Table 1**: Salmonella Cases by Season (2004-2014).

## Data Availability

The data that support the findings of this study are available from the Centers for Disease Control and Prevention (CDC), but restrictions apply to the availability of these data, which are not publicly available. Data are however available from the authors upon reasonable request and with permission of the CDC.

## Abbreviations

CDC: Centers for Disease Control and Prevention
CI: Confidence Interval
FDA: US Food and Drug Administration
FoodNet: Foodborne Diseases Active Surveillance Network
IRR: Incidence Rate Ratio
NOAA: National Oceanic and Atmospheric Administration
PRCP: daily maximum precipitation
TMAX: daily maximum temperature
U.S.: The United States
USDA-FSIS: US Department of Agriculture’s Food Safety

## References

[1] Scallan E, Hoekstra RM, Angulo FJ, Tauxe RV, Widdowson MA, Roy SL (2011). Foodborne illness acquired in the United States-Major pathogens. Emerg Infect Dis.

[2] Chai SJ, White PL, Lathrop SL, Solghan SM, Medus C, McGlinchey BM, et al. Salmonella enterica serotype enteritidis: Increasing incidence of domestically acquired infections. Clin Infect Dis. 2012;54. 10.1093/cid/cis231. 10.1093/cid/cis231 22572674

[3] Greene SK, Daly ER, Talbot EA, Demma LJ, Holzbauer S, Patel NJ (2008). Recurrent multistate outbreak of salmonella newport associated with tomatoes from contaminated fields, 2005. Epidemiol Infect..

[4] Jackson BR, Griffin PM, Cole D, Walsh KA, Chai SJ (2013). Outbreak-associated salmonella enterica serotypes and food commodities, united states, 1998-2008. Emerg Infect Dis..

[5] Tack DM, Marder EP, Griffin PM, Cieslak PR, Dunn J, Hurd S (2019). Preliminary incidence and trends of infections with pathogens transmitted commonly through food — Foodborne Diseases Active Surveillance Network, 10 U.S. sites, 2015–2018. Am J Transplant.

[6] Sanchez S, Hofacre CL, Lee MD, Maurer JJ, Doyle MP (2002). Animal sources of salmonellosis in humans. J Am Vet Med Assoc..

[7] Hohmann EL (2001). Nontyphoidal Salmonellosis. Clin Infect Dis..

[8] Vugia DJ, Samuel M, Farley MM, Marcus R, Shiferaw B, Shallow S (2004). Invasive Salmonella infections in the United States, foodnet, 1996-1999: Incidence, serotype distribution, and outcome. Clin Infect Dis..

[9] Popoff MY, Bockemühl J, Gheesling LL (2004). Supplement 2002 (no. 46) to the Kauffmann-White scheme. Res Microbiol..

[10] Humphrey T (2004). Salmonella, stress responses and food safety. Nat Rev Microbiol..

[11] Jones TF, Ingram LA, Cieslak PR, Vugia DJ, Tobin-D’Angelo M, Hurd S (2008). Salmonellosis outcomes differ substantially by serotype. J Infect Dis..

[12] Akil L, Anwar Ahmad H, Reddy RS (2014). Effects of climate change on Salmonella infections. Foodborne Pathog Dis..

[13] Amuakwa-Mensah F, Marbuah G, Mubanga M (2017). Climate variability and infectious diseases nexus: Evidence from Sweden. Infect Dis Model..

[14] Wu X, Lu Y, Zhou S, Chen L, Xu B (2016). Impact of climate change on human infectious diseases: Empirical evidence and human adaptation. Environ Int..

[15] Cann KF, Thomas DR, Salmon RL, Wyn-Jones AP, Kay D (2013). Extreme water-related weather events and waterborne disease. Epidemiol Infect..

[16] Jiang C, Shaw KS, Upperman CR, Blythe D, Mitchell C, Murtugudde R (2015). Climate change, extreme events and increased risk of salmonellosis in Maryland, USA: Evidence for coastal vulnerability. Environ Int..

[17] Lake IR, Gillespie IA, Bentham G, Nichols GL, Lane C, Adak GK (2009). A re-evaluation of the impact of temperature and climate change on foodborne illness. Epidemiol Infect..

[18] Lal A, Hales S, Kirk M, Baker MG, French NP (2016). Spatial and temporal variation in the association between temperature and salmonellosis in NZ. Aust N Z J Public Health..

[19] Lee D, Chang HH, Sarnat SE, Levy K (2019). Precipitation and salmonellosis incidence in Georgia, USA: Interactions between extreme rainfall events and antecedent rainfall conditions. Environ Health Perspect..

[20] McMichael AJ, Lindgren E (2011). Climate change: Present and future risks to health, and necessary responses. J Intern Med..

[21] Semenza JC, Herbst S, Rechenburg A, Suk JE, Höser C, Schreiber C (2012). Climate change impact assessment of food- and waterborne diseases. Crit Rev Environ Sci Technol.

[22] Stephen DM, Barnett AG (2016). Effect of temperature and precipitation on salmonellosis cases in South-East Queensland, Australia: An observational study. BMJ Open..

[23] IPCC. Climate Change 2014: Synthesis Report. Contribution of Working Groups I, II and III to the Fifth Assessment Report of the Intergovernmental Panel on Climate Change [Internet]. Pachauri, R. K and Meyer LA, editor. Geneva, Switzerland: IPCC; 2014. Available from: https://www.ipcc.ch/site/assets/uploads/2018/02/SYR_AR5_FINAL_full.pdf.

[24] Liu C, Hofstra N, Franz E (2013). Impacts of climate change on the microbial safety of pre-harvest leafy green vegetables as indicated by Escherichia coli O157 and Salmonella spp. Int J Food Microbiol..

[25] Manfreda G, De Cesare A (2016). Novel food trends and climate changes: Impact on emerging food-borne bacterial pathogens. Curr Opin Food Sci..

[26] US Department of Commerce. American community survey, 2010 and 2019 American Community Survey 5-year Estimates [Internet]. Available from: https://data.census.gov/cedsci/table?q=UnitedStates&g=0100000US&tid=ACSDP1Y2018.DP05. Accessed 30 Jan 2021.

[27] USDA. NASS Agricultural Statistics 2017 [Internet]. Washington DC; 2017. Available from: https://www.nass.usda.gov/Publications/AgCensus/2017/index.php#full_report. Accessed 30 Jan 2021.

[28] NOAA. Coastal County Definitions Coastal County Aggregations Coastal Watershed Counties [Internet]. 2017. Available from: https://coast.noaa.gov/data/digitalcoast/pdf/qrt-coastal-county-definitions.pdf. Accessed 30 Jan 2021.

[29] NCDC. The National Climatic Data Center’s Archive of Global Historical Weather and Climate Data [Internet]. NOAA’s Natl. Cent. Environ. Inf. 2020. Available from: https://www.ncdc.noaa.gov/cdo-web/. Accessed 30 Jan 2021.

[30] Romeo Upperman C, Parker J, Jiang C, He X, Murtugudde R, Sapkota A (2015). Frequency of Extreme Heat Event as a Surrogate Exposure Metric for Examining the Human Health Effects of Climate Change. PLoS One..

[31] Byers AL, Allore H, Gill TM, Peduzzi PN (2003). Application of negative binomial modeling for discrete outcomes: A case study in aging research. J Clin Epidemiol..

[32] Greene WH. Accounting for Excess Zeros and Sample Selection in Poisson and Negative Binomial Regression Models [Internet]. Rochester, NY Soc. Sci. Res. Network; 1994 Mar. Rep. No. ID 1293115. Available from: http://ssrn.com/abstract=1293115. Accessed 30 Jan 2021.

[33] Aik J, Heywood AE, Newall AT, Ng LC, Kirk MD, Turner R (2018). Climate variability and salmonellosis in Singapore – A time series analysis. Sci Total Environ..

[34] D’Souza RM, Becker NG, Hall G, Moodie KBA (2004). Does ambient temperature affect foodborne disease?. Epidemiology..

[35] Kovats RS, Edwards SJ, Hajat S, Armstrong BG, Ebi KL, Menne B (2004). The effect of temperature on food poisoning: A time-series analysis of salmonellosis in ten European countries. Epidemiol Infect..

[36] Lake IR. Food-borne disease and climate change in the United Kingdom. Environ Heal A Glob Access Sci Source. 2017;16. 10.1186/s12940-017-0327-0. 10.1186/s12940-017-0327-0 PMC5773878 29219100

[37] Naumova EN, Jagai JS, Matyas B, DeMaria A, MacNeill IB, Griffiths JK (2007). Seasonality in six enterically transmitted diseases and ambient temperature. Epidemiol Infect..

[38] Zhang Y, Bi P, Hiller JE (2012). Projected burden of disease for Salmonella infection due to increased temperature in Australian temperate and subtropical regions. Environ Int.

[39] Powell MR, Crim SM, Hoekstra RM, Williams MS, Gu W (2018). Temporal patterns in principal Salmonella serotypes in the USA; 1996-2014. Epidemiol Infect..

[40] Zhang Y, Bi P, Hiller JE (2010). Climate variations and Salmonella infection in Australian subtropical and tropical regions. Sci Total Environ..

[41] Bambrick HJ, Dear KBG, Woodruff RE, Hanigan IC, McMichael AJ. The impacts of climate change on three health outcomes: temperature-related mortality and hospitalisations, salmonellosis and other bacterial gastroenteritis , and population at risk from dengue. Garnaut Clim Chang Rev. 2008. Available from: http://garnautreview.org.au/CA25734E0016A131/WebObj/03-AThreehealthoutcomes/$File/03-AThreehealthoutcomes.pdf. Accessed 30 Jan 2021.

[42] Boore AL, Hoekstra RM, Iwamoto M, Fields PI, Bishop RD, Swerdlow DL (2015). Salmonella enterica infections in the United States and assessment of coefficients of variation: A Novel approach to identify epidemiologic characteristics of individual serotypes, 1996-2011. PLoS One..

[43] Shaw KS, Cruz-Cano R, Jiang C, Malayil L, Blythe D, Ryan P (2016). Presence of animal feeding operations and community socioeconomic factors impact salmonellosis incidence rates: An ecological analysis using data from the Foodborne Diseases Active Surveillance Network (FoodNet), 2004-2010. Environ Res..

[44] Hadler JL, Clogher P, Libby T, Wilson E, Oosmanally N, Ryan P (2020). Relationship between census tract-level poverty and domestically acquired Salmonella incidence: Analysis of foodborne diseases active surveillance network data, 2010-2016. J Infect Dis..

[45] Milazzo A, Giles LC, Zhang Y, Koehler AP, Hiller JE, Bi P (2016). Heatwaves differentially affect risk of Salmonella serotypes. J Infect..

[46] Srikantiah P, Lay JC, Hand S, Crump JA, Campbell J, Van Duyne MS (2004). Salmonella enterica serotype Javiana infections associated with amphibian contact, Mississippi, 2001. Epidemiol Infect..

[47] Varma JK, Marcus R, Stenzel SA, Hanna SS, Gettner S, Anderson BJ (2006). Highly resistant Salmonella Newport-MDRAmpC transmitted through the domestic US food supply: A FoodNet case-control study of sporadic Salmonella Newport infections, 2002-2003. J Infect Dis..

[48] Shi X, Namvar A, Kostrzynska M, Hora R, Warriner K (2007). Persistence and growth of different Salmonella serovars on pre- and postharvest tomatoes. J Food Prot..

[49] Hoelzer K, Isabel A, Switt M, Wiedmann M (2011). Hoelzer 2011 animal contact as a source of human non-typhoidal salmonellosis. Vet Res..

[50] Graham JP, Nachman KE (2010). Managing waste from confined animal feeding operations in the United States: The need for sanitary reform. J Water Health..

[51] Smith JE, Perdek JM (2004). Assessment and Management of Watershed Microbial Contaminants. Crit Rev Environ Sci Technol..

[52] Bell RL, Zheng J, Burrows E, Allard S, Wang CY, Keys CE (2015). Ecological prevalence, genetic diversity, and epidemiological aspects of Salmonella isolated from tomato agricultural regions of the Virginia Eastern Shore. Front Microbiol..

[53] Micallef SA, Rosenberg Goldstein RE, George A, Kleinfelter L, Boyer MS, McLaughlin CR (2012). Occurrence and antibiotic resistance of multiple Salmonella serotypes recovered from water, sediment and soil on mid-Atlantic tomato farms. Environ Res..

[54] Ge C, Lee C, Lee J (2012). The impact of extreme weather events on Salmonella internalization in lettuce and green onion. Food Res Int..

[55] Matthews L, McKendrick IJ, Ternent H, Gunn GJ, Synge B, Woolhouse MEJ (2006). Super-shedding cattle and the transmission dynamics of Escherichia coli O157. Epidemiol Infect..

[56] Pangloli P, Dje Y, Ahmed O, Doane CA, Oliver SP, Draughon FA (2008). Seasonal incidence and molecular characterization of Salmonella from dairy cows, calves, and farm environment. Foodborne Pathog Dis..

[57] Curriero FC, Patz JA, Rose JB, Lele S (2001). The association between extreme precipitation and waterborne disease outbreaks in the United States, 1948-1994. Am J Public Health..

[58] Eisenhauer IF, Hoover CM, Remais JV, Monaghan A, Celada M, Carlton EJ (2016). Estimating the risk of domestic water source contamination following precipitation events. Am J Trop Med Hyg..

[59] Gershunov A, Benmarhnia T, Aguilera R (2018). Human health implications of extreme precipitation events and water quality in California, USA: a canonical correlation analysis. Lancet Planet Heal.

[60] Rose JB, Epstein PR, Lipp EK, Sherman BH, Bernard SM, Patz JA (2001). Climate variability and change in the United States: potential impacts on water- and foodborne diseases caused by microbiologic agents. Environ Health Perspect..

[61] Alegbeleye OO, Singleton I, Sant’Ana AS (2018). Sources and contamination routes of microbial pathogens to fresh produce during field cultivation: A review. Food Microbiol..

[62] Holvoet K, Sampers I, Seynnaeve M, Uyttendaele M (2014). Relationships among hygiene indicators and enteric pathogens in irrigation water, soil and lettuce and the impact of climatic conditions on contamination in the lettuce primary production. Int J Food Microbiol..

[63] Clarkson LS, Tobin-D’angelo M, Shuler C, Hanna S, Benson J, Voetsch AC (2010). Sporadic Salmonella enterica serotype Javiana infections in Georgia and Tennessee: A hypothesis-generating study. Epidemiol Infect..

[64] Mukherjee N, Nolan VG, Dunn JR, Banerjee P (2019). Sources of human infection by Salmonella enterica serotype Javiana: A systematic review. PLoS One..

[65] Huang JY, Patrick ME, Manners J, Sapkota AR, Scherzinger KJ, Tobin-D’Angelo M (2017). Association between wetland presence and incidence of Salmonella enterica serotype Javiana infections in selected US sites, 2005-2011. Epidemiol Infect..

[66] USGS. National water summary-Wetland Resources: Management and Research [Internet]. U.S. Geol. Surv. Water-Supply Pap. 2425 [Compiled by Fretwell JD, Williams JS, Redman PJ] U.S. Gov. Print. Off. Washingt. DC. 1996. 10.3133/wsp2425

[67] USDA. 2017 Census of Agriculture: United States Summary and State Data, Vol. 1 [Internet]. Geogr. Area Ser. Part 51. U.S. Dep. Agric. Natl. Agric. Stat. Serv. 2019. Available from: https://www.nass.usda.gov/Publications/AgCensus/2017/Full_Report/Volume_1,_Chapter_1_US/usv1.pdf. Accessed 30 Jan 2021.

[68] CT DPH. Private Well Program Report [Internet]. Connect. State Dep. Public Heal. Available from: https://portal.ct.gov/dph/Environmental-Health/Private-Well-Water-Program/Private-Wells. Accessed 30 Jan 2021.

[69] Dieter, C.A., Maupin, M.A., Caldwell, R.R., Harris, M.A., Ivahnenko, T.I., Lovelace, J.K., Barber, N.L., and Linsey KS. Estimated use of water in the United States in 2015: U.S. Geological Survey Circular 1441. [Supersedes USGS Open-File Rep. 2017–1131]. 2018.

[70] Dev Kumar G, Williams RC, Sriranganathan N, Boyer RR, Eifert JD (2018). Survival of tomato outbreak associated salmonella serotypes in soil and water and the role of biofilms in abiotic surface attachment. Foodborne Pathog Dis..

[71] Underthun K, De J, Gutierrez A, Silverberg R, Schneider KR (2018). Survival of salmonella and Escherichia coli in two different soil types at various moisture levels and temperatures. J Food Prot..

[72] MD Department of State Planning. Natural soil groups of Maryland, generalized land use plan. Baltimore, MD. Publication:199 [Internet]. 1973. Available from: https://planning.maryland.gov/Documents/OurProducts/Publications/OtherPublications/soil_group_of_md.pdf. Accessed 30 Jan 2021.

[73] Hellberg RS, Chu E (2016). Effects of climate change on the persistence and dispersal of foodborne bacterial pathogens in the outdoor environment: A review. Crit Rev Microbiol..

[74] Meerburg BG, Singleton GR, Kijlstra A. Rodent-borne diseases and their risks for public health Rodent-borne diseases and their risks for public health. Crit. Rev. Microbiol. 2009. 10.1080/10408410902989837. 10.1080/10408410902989837 19548807

[75] Mills JN, Gage KL, Khan AS (2010). Potential influence of climate change on vector-borne and zoonotic diseases: A review and proposed research plan. Environ Health Perspect..

