# Supplementary material for: Climate change, extreme events, and increased risk of salmonellosis: foodborne diseases active surveillance network (FoodNet), 2004-2014
Source: Environ Health. 2021 Sep 18;20:105. doi: 10.1186/s12940-021-00787-y (PMC8449873; doi:10.1186/s12940-021-00787-y)
Supplement: Supplementary file 1 — Additional file 1: Supplemental Table 1: Salmonella Cases by Season (2004-2014). [file 12940_2021_787_MOESM1_ESM.docx]

Supplemental Table 1: Salmonella Cases by Season (2004-2014)

| Cases | | Winter | Spring | Summer | Fall |  |
| --- | --- | --- | --- | --- | --- | --- |
| Serovar* | |  |  |  |  |  |
|  | Enteritidis | 1890 | 2649 | 3747 | 2367 |  |
|  | Javiana | 409 | 352 | 2578 | 2470 |  |
|  | Newport | 608 | 699 | 3452 | 2696 |  |
|  | Typhimurium | 1574 | 1499 | 3163 | 2801 |  |
| Avg Temp**(°C) | | 0.7 | 11.0 | 21.8 | 12.4 |  |
| Avg Precip**(mm) | | 152.1 | 224.5 | 220.9 | 214.9 |  |
| *Centers for Disease Control and Prevention data for the 7 FoodNet sites analyzed (Connecticut, Georgia, Maryland, Minnesota, New Mexico, Oregon, and Tennessee) | | | | | |  |
|  |  |  |  |  |  |  |
| **Based on data from NOAA's National Centers for Environmental Information | | | | | |  |
